# Supplementary material for: Modeling glioblastoma heterogeneity as a dynamic network of cell states
Source: Mol Syst Biol. 2021 Sep 16;17(9):e10105. doi: 10.15252/msb.202010105 (PMC8444284; doi:10.15252/msb.202010105)
Supplement: Supplementary file 6 — Source Data for Figure 5 [file MSB-17-e10105-s004.zip › Figure5A_sourcedata/GSEA_3017/hallmarks_stateF.GseaPreranked.1621934628066/HALLMARK_UV_RESPONSE_UP.html]

Details for gene set HALLMARK\_UV\_RESPONSE\_UP[GSEA]

|  || Dataset | state33017 |
| Phenotype | NoPhenotypeAvailable |
| Upregulated in class | na\_neg |
| GeneSet | HALLMARK\_UV\_RESPONSE\_UP |
| Enrichment Score (ES) | -0.21414943 |
| Normalized Enrichment Score (NES) | -1.0099422 |
| Nominal p-value | 0.43736264 |
| FDR q-value | 0.5523699 |
| FWER p-Value | 1.0 |
Table: GSEA Results Summary

  

Fig 1: Enrichment plot: HALLMARK\_UV\_RESPONSE\_UP      
 Profile of the Running ES Score & Positions of GeneSet Members on the Rank Ordered List

  

| PROBE | GENE SYMBOL | GENE\_TITLE | RANK IN GENE LIST | RANK METRIC SCORE | RUNNING ES | CORE ENRICHMENT || 1 | SQSTM1 |  |  | 54 | 0.914 | 0.0378 | No |
| 2 | BTG2 |  |  | 124 | 0.708 | 0.0550 | No |
| 3 | HMOX1 |  |  | 142 | 0.684 | 0.0938 | No |
| 4 | BTG1 |  |  | 222 | 0.574 | 0.0973 | No |
| 5 | CA2 |  |  | 243 | 0.558 | 0.1262 | No |
| 6 | TFRC |  |  | 270 | 0.537 | 0.1511 | No |
| 7 | IL6ST |  |  | 321 | 0.500 | 0.1626 | No |
| 8 | ALDOA |  |  | 377 | 0.479 | 0.1704 | No |
| 9 | EPHX1 |  |  | 456 | 0.448 | 0.1657 | No |
| 10 | FOS |  |  | 592 | 0.401 | 0.1322 | No |
| 11 | NTRK3 |  |  | 621 | 0.391 | 0.1463 | No |
| 12 | ATP6V1F |  |  | 685 | 0.374 | 0.1434 | No |
| 13 | SLC25A4 |  |  | 836 | 0.340 | 0.0990 | No |
| 14 | ATP6V1C1 |  |  | 1053 | 0.299 | 0.0222 | No |
| 15 | NPTXR |  |  | 1168 | 0.283 | -0.0099 | No |
| 16 | TAP1 |  |  | 1317 | 0.266 | -0.0584 | No |
| 17 | GRINA |  |  | 1383 | 0.259 | -0.0701 | No |
| 18 | JUNB |  |  | 1466 | 0.251 | -0.0899 | No |
| 19 | LHX2 |  |  | 1597 | -0.285 | -0.1290 | No |
| 20 | PARP2 |  |  | 1665 | -0.305 | -0.1384 | No |
| 21 | PDAP1 |  |  | 1758 | -0.343 | -0.1565 | No |
| 22 | RFC4 |  |  | 1887 | -0.413 | -0.1861 | Yes |
| 23 | POLE3 |  |  | 1901 | -0.426 | -0.1630 | Yes |
| 24 | FKBP4 |  |  | 1903 | -0.426 | -0.1344 | Yes |
| 25 | EIF5 |  |  | 1923 | -0.439 | -0.1131 | Yes |
| 26 | STIP1 |  |  | 1933 | -0.446 | -0.0868 | Yes |
| 27 | AGO2 |  |  | 1966 | -0.462 | -0.0698 | Yes |
| 28 | PSMC3 |  |  | 1999 | -0.492 | -0.0508 | Yes |
| 29 | FEN1 |  |  | 2018 | -0.511 | -0.0241 | Yes |
| 30 | GGH |  |  | 2080 | -0.603 | -0.0106 | Yes |
| 31 | HNRNPU |  |  | 2090 | -0.628 | 0.0281 | Yes |
| 32 | DDX21 |  |  | 2107 | -0.659 | 0.0657 | Yes |
Table: GSEA details [plain text format]

  

Fig 2: HALLMARK\_UV\_RESPONSE\_UP: Random ES distribution      
 Gene set null distribution of ES for **HALLMARK\_UV\_RESPONSE\_UP**
